# Hybrid Dirac semimetal-based photodetector with efficient low-energy photon harvesting

**DOI:** 10.1038/s41377-022-00741-8

**Published:** 2022-03-10

**Authors:** Lin Wang, Li Han, Wanlong Guo, Libo Zhang, Chenyu Yao, Zhiqingzi Chen, Yulu Chen, Cheng Guo, Kaixuan Zhang, Chia-Nung Kuo, Chin Shan Lue, Antonio Politano, Huaizhong Xing, Mengjie Jiang, Xianbin Yu, Xiaoshuang Chen, Wei Lu

**Affiliations:** 1 State Key Laboratory for Infrared Physics, Shanghai Institute of Technical Physics, Chinese Academy of Sciences, 500 Yu-tian Road, Shanghai, 200083 China; 2 Department of Optoelectronic Science and Engineering, Donghua University, Shanghai, 201620 China; 3 College of Physics and Optoelectronic Engineering, Hangzhou Institute for Advanced Study, University of Chinese Academy of Sciences, No. 1, Sub-Lane Xiangshan, Xihu District, Hangzhou, 310024 China; 4 School of Physical Science and Technology, ShanghaiTech University, Shanghai, 201210 China; 5 The 50th Research Institute of China Electronics Technology Group, Shanghai, 200331 China; 6 Research Center for Intelligent Network, Zhejiang Lab, Hangzhou, 311121 China; 7 Department of Physics, Cheng Kung University, 1 Ta-Hsueh Road, 70101 Tainan, Taiwan China; 8 INSTM and Department of Physical and Chemical Sciences, University of L’Aquila, via Vetoio, 67100 L’Aquila (AQ), Italy; 9 CNR-IMM Istituto per la Microelettronica e Microsistemi, VIII strada 5, I-95121 Catania, Italy

**Keywords:** Optical materials and structures, Electronics, photonics and device physics

## Abstract

Despite the considerable effort, fast and highly sensitive photodetection is not widely available at the low-photon-energy range (~meV) of the electromagnetic spectrum, owing to the challenging light funneling into small active areas with efficient conversion into an electrical signal. Here, we provide an alternative strategy by efficiently integrating and manipulating at the nanoscale the optoelectronic properties of topological Dirac semimetal PtSe_2_ and its van der Waals heterostructures. Explicitly, we realize strong plasmonic antenna coupling to semimetal states near the skin-depth regime (λ/10^4^), featuring colossal photoresponse by in-plane symmetry breaking. The observed spontaneous and polarization-sensitive photocurrent are correlated to strong coupling with the nonequilibrium states in PtSe_2_ Dirac semimetal, yielding efficient light absorption in the photon range below 1.24 meV with responsivity exceeding ∼0.2 A/W and noise-equivalent power (NEP) less than ~38 pW/Hz^0.5^, as well as superb ambient stability. Present results pave the way to efficient engineering of a topological semimetal for high-speed and low-energy photon harvesting in areas such as biomedical imaging, remote sensing or security applications.

## Abstract (web summary)

A versatile manipulation of the optoelectronic properties within the Dirac-semimetals and its van der Waals heterostructures beyond skin-depth limit, opening up substantial potential for engineering spontaneous photocurrent with high SNR ratio and imaging at far-IR/THz band at room temperature.

## Graphical abstract

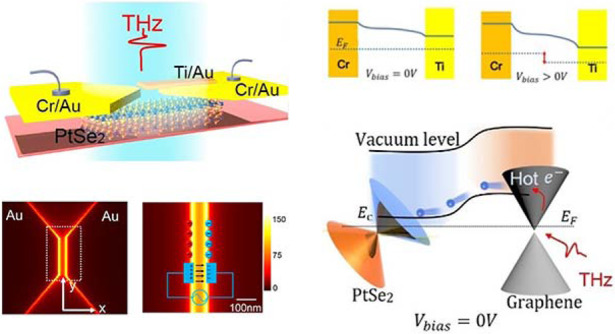

## Introduction

Leapfrog progress of optoelectronic devices, including the demonstration of a super-steep-slope transistor enabled by exotic optical and electronic properties, have stimulated widespread pursuit of van der Waals (vdW) combination and stacking order with peculiar band-structure and suitable optoelectronic capabilities for addressing practical requirements of advanced optical communications and sensing technologies^1–3^. The broad family of transition-metal dichalcogenides (MX_2_, with M being metal and X standing for S, Se, or Te) represents a suitable platform with unique physical properties and functionalities complementary to those of graphene^4–7^. Especially, photovoltaic devices based on vdW heterostructures with peculiar band-alignment own integrated properties revolutionizing early landmarks of photonic technologies in the visible or near-infrared regimes^8,9^. Nevertheless, the main technological bottlenecks of photodetection lie in the low-photon-energy range of the electromagnetic spectrum, since the detectable wavelength is exacerbated not only by the selective bandgap dependence but also by the complexity regarding the efficient conversion of photon energy into an electrical signal within a small nanoscale active area. To overcome state-of-the-art limitations impeding strong light-matter interaction, the assessment of novel materials with unusual electronic properties is critical.

Among the broad class of transition-metal dichalcogenides, platinum diselenide (PtSe_2_) provides the unique advantages of broadband light absorption, due to the presence of type-II Dirac fermions^10,11^. High-efficiency photoelectric conversion in the visible and infrared bands has been initially verified in PtSe_2_ and related Van der Waals phototransistors^12–14^. Therefore, PtSe_2_ represents a suitable candidate for implementing innovative optoelectronic devices exploiting the peculiar topology of its energy bands and the corresponding Berry-curvature induced phenomena. Recently, the divergent bulk photovoltaic effect has been observed in Weyl-type topological semimetals without inversion symmetry, stemming from the shift of charge center or the spontaneous current, due to the divergence of Berry-flux field in the vicinity of Weyl nodes^15,16^. Though semimetals-based photodetectors are affected by inevitable drawbacks related to the high dark current^17^, the topological nature of their electronic bands holds great promise for the development of broadband photoresponse down to the terahertz (THz) band to circumvent technology bottlenecks^18–22^.

In this blossoming field, PtSe_2_ topological Dirac semimetal, with its versatile symmetry-breaking operation, provides unprecedented opportunities to explore Dirac fermiology beyond early landmarks of graphene-based optoelectronics. Especially, in the prospect of device applications, understanding the leading role of nonequilibrium states, especially at the low-energy THz band, is indispensable to achieve efficient charge separation crucial to address technology-oriented issues. In this work, the unusual photoconductive behavior in PtSe_2_ and its van der Waals integration provides a versatile platform towards the synergistic manipulation of optoelectronic capabilities for high-performance THz detection via geometrical and band-structure engineering. We implement optically excited hot electrons under ultrastrong interaction with near-field in the skin-depth regime, followed by the quasi-equilibrium transport obliterating the drawbacks imposed by power-hungry, with the reduction of thermal-agitation noise and the achievement of chip-level integration for low-energy photodetection.

## Results

### Plasmonic antenna coupling toward the skin-depth limit

**Fig. 1:**
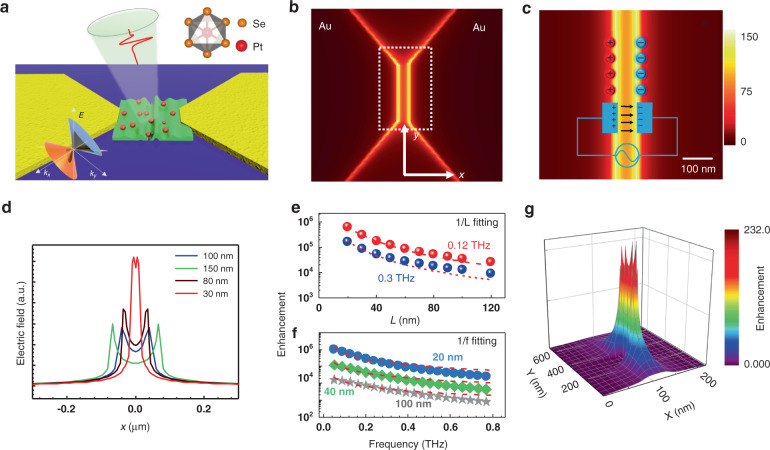
Scaling law of plasmonic near-field characteristic toward the skin-depth regime. **a** Schematics of the bow-tie antenna-assisted device. **b**, **c** The cross-section view of the simulated electric field intensity normalized to incident one marks the power-gain around the nanochannel at 0.3 THz electromagnetic waves. Localized oscillating electric field enhancement in the sub-100 nm channel by light-induced current charges. **d** The profile of electric field distribution along the x-axis. **e**, **f** The scaling of electric field enhancement derived from FDTD method versus channel length and incident frequency. **g** The spatial distribution of the electric field near the 100 nm gapped channel substantiates the strong focusing induced by the oscillating charge

PtSe_2_ crystallizes in a centrosymmetric CdI_2_-type structure with space group P$\bar 3$m1. Pt atoms occupy the octahedral sites in alternate Se layers, while the adjacent unoccupied Se layers are held together by weak van der Waals forces^23,24^. PtSe_2_ crystals, grown by chemical vapor transport method, show superb cleanliness, as demonstrated by the survey spectrum acquired by X-ray photoemission spectroscopy (XPS) in Figure S1 (Supporting Information), in which only Pt- and Se-derived core levels are present, contrary to other studies in which strong contamination by C and O were reported^25,26^. As demonstrated in ref. ^10^, PtSe_2_ hosts type-II Dirac bulk states featuring tilted Dirac-cone dispersion breaking Lorentz invariance, providing an ideal candidate to investigations of relativistic quasiparticles in condensed-matter systems (Fig. 1a). It has also been discovered that undamped plasmons and large absorption cross-section at terahertz frequency emerge for materials that possess type-II Dirac cone^27^. The presence of low-energy quasiparticles enables unique properties, such as anisotropic thermoelectricity and high room-temperature mobility (higher than 1800 cm^2^/V·s)^28^. We have also measured the transfer curve of the semiconducting PtSe_2_ device was with a 5 nm thick flake, and its mobility is approaching 965 cm^2^/(V·s) (Figure S2, Supporting Information). Accordingly, semimetal PtSe_2_ represents a suitable candidate for low-energy photodetection. However, unlike the visible/near-infrared region, where the intrinsic plasmons of metallic nanoparticles with distinct field-coupling can be deployed and be commensurate with the small active channel^29^, the delocalization of electromagnetic field and large mismatch in dimension at the low-energy THz regime inhibits the efficient light absorption.

To validate the capability of optical-field enhancement, a bow-tie antenna with a subwavelength gap is modeled by utilizing the finite difference time domain method (FDTD), in which the photoactive region varies from 30 to 100 nm in the metallic nanoslit (Fig. 1b, c). The specific simulation details are given in the Supporting Information. Qualitatively, the figure of an effective line-capacitor driven by light-induced alternating currents delineates that the electric field intensity will keep growing even whenever the gap size shrinks toward the skin-depth limit, as depicted in Figs. 1d, e. Consequently, THz spoof-plasmon polaritons can be efficiently launched near the metal-PtSe_2_ interface, which will convert an incident electromagnetic field into the localized oscillating electric field. Figure 1f shows the continuous optical-field enhancement by effectively focusing THz waves in the λ/30000 metal slit^29^ via decreasing gap-slit widths towards the skin-depth limit. We argue that this mode, featuring very strong optical absorption, plays a crucial role in the experimental realization of highly efficient low-energy photon absorption and detection at the nanoscale active area by introducing an ultra-sharp gap in combination with the intentional breaking of in-plane symmetry (Fig. 1g).

### Device operational principle and photocurrent characterization

**Fig. 2:**
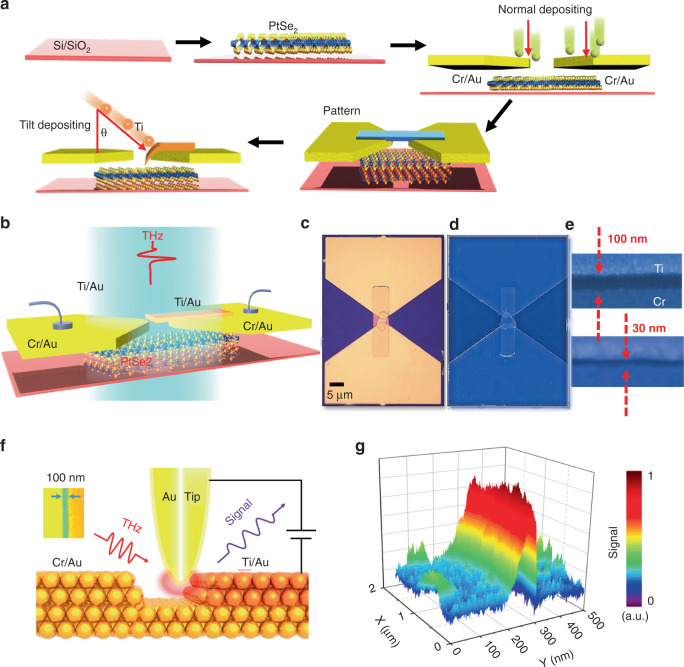
The nanogap slit fabrication technology and s-SNOM. **a** The nanogap slit device fabrication process, including PtSe_2_ fabrication, ultraviolet lithography, lift-off processes, and tilt deposition. **b** Schematic of the bow-tie antenna-assisted device. **c**–**e** Optical microscopy and false-color SEM images of the PtSe_2_ nanogap slit device with a typical channel length (100 nm and 30 nm). **f** The near-field images are taken around the slit area using broadband illumination. **g** Stereograph of the near-field signal

To validate a proof-of-concept, we engineer different active channels based on PtSe_2_ nanosheets (see “Materials and methods” for details on the fabrication of the microgap slit detectors) by using the tilt-angle method. Since PtSe_2_ will change from metallic to semiconductor states as the thickness decreases^30^, we mainly focus on the bulk PtSe_2_ flake of above 30 nm, which can be regarded as an example of type-II Dirac semimetal. The Cr/Au contacts of two electrodes are evaporated in advance onto the PtSe_2_ surface, which is defined by electron beam lithography (EBL) resulting in a 4 μm channel. To obtain the nanogap between the two electrodes, the channel regions are self-aligned by EBL and then evaporated now with Ti/Au again under the different tilt angle (30° < *θ* <70 °) in Fig. 2a, b, to ensure the in-plane asymmetry for the unilateral flow of the nonequilibrium carriers. Our strategy overcomes the limitation of light absorption, and enables spontaneous photocurrent, due to the asymmetric metal-material interfaces. The resulting device is represented in Fig. 2c–e. The typical channel lengths (*L*) can be tuned from 30 to 100 nm, allowing the contact of the PtSe_2_ nanosheets with two asymmetrical electrodes. To prove the efficiency of light-focusing towards the photoactive area, we investigate the formation of field enhancement by using scattering-type scanning near-field optical microscope (s-SNOM) (Fig. 2f). The asymmetrical device shows a significant near-field scattering signal concentrating near the small active area (Fig. 2g), thus achieving a combination with strong light-matter interaction in the scale of λ/10^4^ and intentional breaking of in-plane symmetry, which supports the feasibility of efficient low-energy photodetection.

**Fig. 3:**
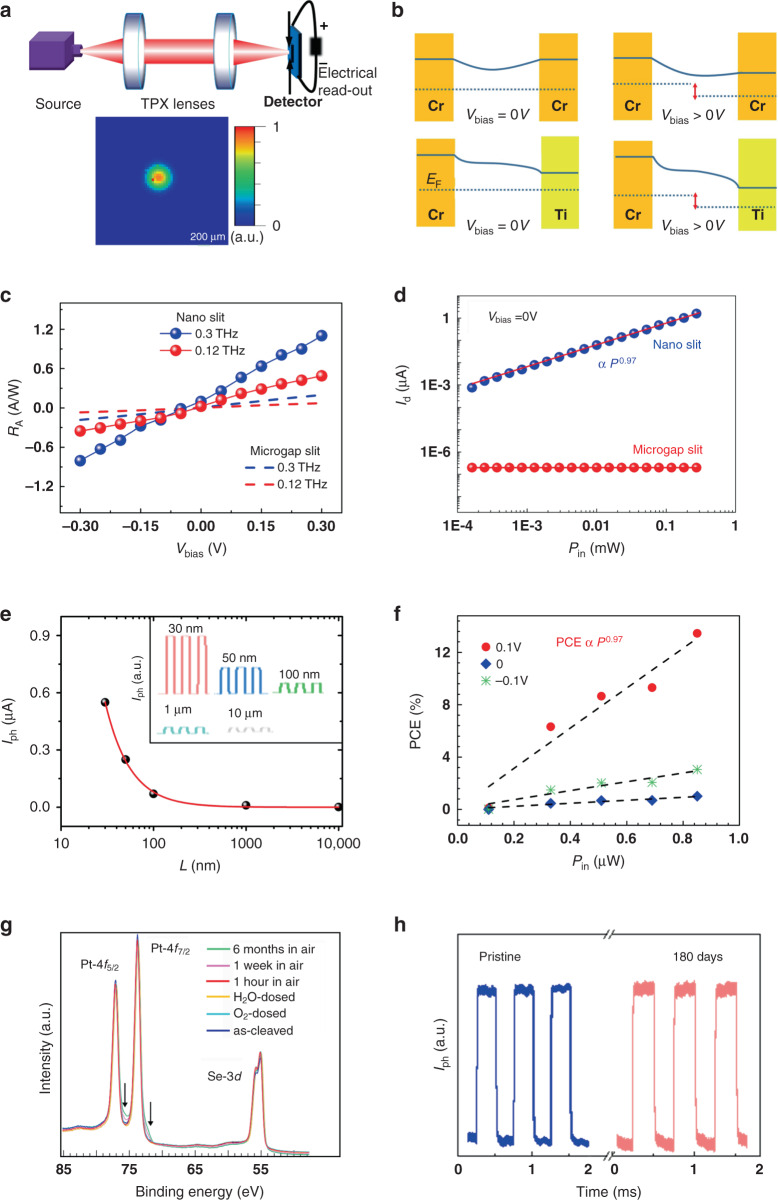
Characteristics of the PtSe2 low-energy photon detector. **a** Schematic diagram of the experimental geometry, with photon-beam spot profile (diameter: 800 μm), derived from two-dimensional scanning of the photodetector. **b** The band diagram at the different metal junction regions with/without bias. **c** Bias dependence of responsivity at 0.12 THz and 0.3 THz for different channel lengths. **d** The measured photocurrent vs. output power *P*_in_, with the power, varied from 0.3 μW to 300 μW. The channel length of the typical microgap slit and nanoslit device is 4 μm and 100 nm, respectively. **e**, **f** The conversion efficiency of the nanogap slit photodetector versus the incident power at room temperature. **g** Pt-4f and Se-3d core levels, measured at a photon energy of 400 eV. **h** Time-resolved photo signal of PtSe_2_-based nanogap slit photodetector at *V*_bias_ = 0 V

For the better assessment of gap-size dependent performance, we measure the responsivity (*R*_*A*_ = *I*_*ph*_*R*/(*PS*), where *P* is the power density (0.12 THz: *P* = 10 μW/mm^2^) and the *S* is the diffraction-limited area *S* = *S*_*λ*_ = *λ*^*2*^/*4*) of all devices, by implementing a tunable microwave oscillator and VDI diode-based frequency multiplier for producing incident electromagnetic waves from 0.1 to 0.3 THz (0.41–1.24 meV) (see “Materials and methods” for a detailed description of the experimental setup). The incoming THz beam is collimated and focused into a circular spot by two polymethyl pentene (TPX) lenses with the spatial intensity profile depicted in Fig. 3a. The near-field produced within the antenna efficiently launches the local nonequilibrium-electron distribution, mediated mostly by the intraband transition with randomized momentum-distribution at the PtSe_2_-electrode interface, and, meanwhile, the large density of states originating from non-closing electron-hole pockets in the type-II Dirac semimetal enables its large absorption at THz frequencies^31^. These nonequilibrium states can be accelerated unilaterally from one side to another side of the photoactive area, following the applied static electric field, whereas the spontaneous photocurrent is feasible through breaking the in-plane symmetry, following the tilt-angle method (see Figure S3 in Supporting Information).

Figure 3c (the dotted lines) shows the curves of the responsivity (*R*_A_) of the microgap slit device with the same electrodes, recorded as a function of bias voltage (*V*_bias_) at room temperature. Note that the change of responsivity is almost linear concerning the bias voltage, without saturation even under a high bias, substantiating the photoconductive mechanism^32^. In addition, the linear increase in photocurrent with respect to *V*_bias_ is probably a hint of the bolometric effect. Therefore, we conduct the variable temperature test, as the temperature increases, the device resistance increases. On the contrary, the output current increases significantly under THz radiation heating when the device is biased at positive or negative voltages, which directly rules out the bolometric origin (see Figure S3 in Supporting Information). To gain full insight into the bias effect, we also fabricate devices with crossed-arranged electrodes (see Figure S4 in Supporting Information). The bias-dependent behavior of different electrode pairs by changing the incident photon shows consistent results supporting the bias-induced asymmetry for nonequilibrium hot carriers (Figure S3, Supporting Information), as is also shown in ref. ^33^. To discuss the technological relevance of our photodetector, we then characterize the timescale of our device under the TTL modulated radiation switch. To efficiently extract the 3 dB electrical bandwidth, the time required for the photocurrent to increase from 10 to 90% on the rising edge or, analogously, on the falling edge of a single pulse is defined as the rise or fall time, respectively. Our detector yields a rise time (*τ*_r_) of 1 µs and decay time (*τ*_d_) of 1.8 µs, validating the real-time imaging capability (Figure S5 in Supporting Information)^34^. Despite these advantages, semimetal-based photodetectors require an unbiased operation to reduce the dark current traversing across the active region when the bias voltage is applied. Nevertheless, in contrast to semiconductor-based detectors, preferential flow cannot be achieved through the reversed bias approach for both photoexcited charge-separation and dark current suppression, due to the weakness of semimetal-induced junction, making it a big challenge by simply following the routes of the traditional photon-type detector^17^. As a consequence, microgap slit detectors operating in an unbiased mode need to achieve charge directional migration through other mechanisms, such as (i) a built-in electric field, (ii) the photo-thermoelectric effect, or (iii) the photo-Dember effect^35–37^. The short transient lifetime of photoexcited carriers in semimetals further aggravates the preferential flow charge-separation, making it rather inefficient with a subsequent unsatisfactory photodetection responsivity^17^.

To gain further insight into the behavior of PtSe_2_-based nanogap slit devices (asymmetrical electrodes) with asymmetric metallization, we report the typical output characteristics in Figure S6 of the Supporting Information. Our design obliterates the above adverse effects, by breaking the mirror symmetry of the built-in potential profile (Fig. 3b) and the time window for charge separation. The asymmetrical electrodes forming the nanoscale photoactive region can funnel efficiently the low-energy photons and enable intensive field enhancement, giving rise to a Seebeck electromotive force and a preferential flow of nonequilibrium hot carriers^38^. Furthermore, the conceptual design is also applicable to other layered systems such as Weyl or Moiré superlattice possessing intrinsic inversion-symmetry breaking or quantum shift current term^39^. Remarkably, the spontaneous photocurrent at zero voltage is significantly larger by introducing an asymmetrical contact, with an improvement in sensitivity, reaching a responsivity as high as 0.2 A/W at 0.3 THz (Fig. 3d). The occurrence of a large dynamic range is demonstrated by using a power-tunable electromagnetic source generated from Agilent E8257D (“Materials and methods”). The behavior of the photocurrent *I*_ph_ indicates that *I*_ph_ ∝ *P*_in_^*α*^ with *α* = (0.97 ± 0.005) with the incident power *P*_in_ (Fig. 3c) tuned from 0.3 to 300 μW. Thus, the photocurrent depends linearly on the incident power over a large range of more than three orders of magnitude, similar to the case of graphene-based terahertz devices^34^. Here, by comparing the performance of nanogap slit devices in Fig. 3e, we find that when the channel length approaches the skin limit, the photocurrent reaches a peak due to the intensive field enhancement and reduced probability of carrier scattering near the skin-depth regime. By evaluating the equivalent power of the converted electrical signal, the maximum conversion efficiency of 13.5% (PCE = *P*_e_/*P*_in_) is found. Even though this value is trivial for reported solar cells such as CsPbI_3_ or many others^40^, it is prominent at the terahertz band because of the less efficient light-induced charge-separation effect. These findings also reveal that a strong THz near-field at metal-channel interface triggers nonequilibrium carriers efficiently for photocurrent production, and the even higher photocurrent is expected by traversing bias or improving further the device structure.

It is worth mentioning that the long-term stability of two-dimensional materials remains an important concern for device reliability. Figure S7a (Supporting Information) shows Raman spectra of the pristine PtSe_2_ and the same sample after storage in air for six months. The tiny differences in the Raman spectrum of the aged PtSe_2_ samples suggest their good ambient stability, fully validated by the combination of surface-science experiments. Explicitly, to assess the chemical reactivity of PtSe_2_ toward ambient gases, we expose the as-cleaved surface to doses as high as 10^6^ L of O_2_ and H_2_O in vacuum conditions (1 L = 10^−6^ Torr·s) and we measure core levels by X-ray photoelectron spectroscopy, carried out with synchrotron radiation to enhance surface sensitivity and to improve the energy resolution to follow core-level shifts (Fig. 3g). To test ambient stability, we keep the sample in the air for different periods, from 1 h to 6 months. Even prolonged exposure to air did not induce any change in Se-3d core levels. Only Pt-4f is slightly modified, with the emergence of a doublet with the *J* = 7/2 component at 72.1 eV (see fit in Supporting Information, Figure S7) due to the adsorption of CHx groups from airborne contamination at Se vacancies at the surface of PtSe_2_^41^, with a saturation coverage below 0.05 ML (with ML being monolayer, see vibrational spectra in Figure S7 of the Supporting Information). Correspondingly, the long-term time-resolved photo signal of PtSe_2_-based photodetector is also recorded. The negligible photocurrent decrease enables by the air stability of PtSe_2_ contrasts with the rapid deterioration experienced in the case of black phosphorus or silicene^42,43^.

### Spontaneous photocurrent derived from vdW integration, sensitivity, and device benchmark

**Fig. 4:**
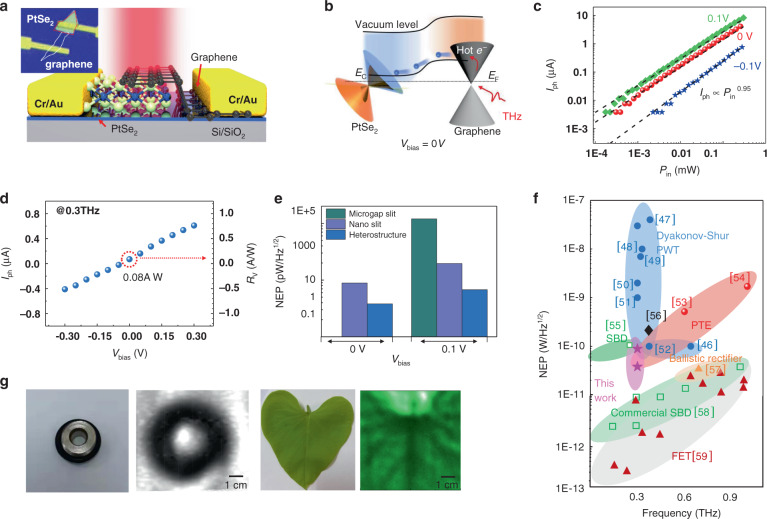
Characteristics of the PtSe2/graphene low-energy photon detector. **a** Schematic diagram of the device architecture. Inset: the optical micrograph of the device. **b** Schematic band diagram of PtSe_2_-graphene heterostructure. **c** The measured photocurrent vs. incident power *P*_in_ with the power varied from 0.3 μW to 300 μW. **d** Bias dependence of photocurrent at 0.3 THz, yielding responsivity of 0.08 A/W at unbiased voltage. **e** Comparisons of NEP at 0.3 THz among pristine metal-PtSe_2_-metal, asymmetric nanogap slit device, and PtSe_2_-graphene vdW heterostructure. **f** Summary of the measurement of our devices and comparison with past works. PTE = photo-thermoelectric effect, Dyakonov-Shur PWT = Dyakonov-Shur plasma wave theory, SBD = Schottky barrier diode, FET = Field-Effect Transistors. Black phosphorus (BP)/hBN heterostructure (ref. ^45^), BP FET (ref. ^47^), Bi_2_Te_(3−x)_Se_x_ FET (ref. ^48^), BP nano-transistors (ref. ^49^), Nanowire (NW) Field-Effect Transistors (ref. ^50^), Se-doping InAs NWs FETs (ref. ^51^), Hg_1–x_Cd_x_Te (ref. ^52^), CVD Graphene (ref. ^53^), Antenna Enhanced Graphene (ref. ^54^), InGaAs Schottky barrier diode (SBD) array (ref. ^55^), Graphene ballistic rectifier (ref. ^56^), Commercial Golay (ref. ^57^), Commercial VDI (ref. ^46^), SBD FETs (ref. ^58^). The pick represents the performance of nanoslit and heterostructure devices, respectively. **g** The optical picture of a fresh leaf and a metallic ring used in the imaging and 0.3 THz transmissions nondestructive image of the fresh leaf demonstrates the leaf vein, which partly contains more moisture

From the above-mentioned argumentations, an in-plane electric polarization can be introduced by symmetry engineering through the work function of metal contacts or the doping level of a semimetal. Although the hybridization of van der Waals heterostructures can lead to the flexible control of in-plane or out-of-plane polarization with upgraded property over segregated parts, there are inherent differences from reverse bias semiconductor-based PN schemes in the photon-type device^9^, which are ascribed to (i) the inefficient low-photon-energy excitation and (ii) the large interface barrier impeding the intraband carrier transport. To reduce the effect of thermal-agitation noise, and in the meantime, to improve the ability of unbiased operation, we consider the possibility of integrating two-dimensional PtSe_2_ with other low-barrier materials. In this perspective, the exploitation of PtSe_2_-graphene heterostructures is envisioned for low-energy photon detection as a promising route to circumvent the excess dark noise. The assembly of PtSe_2_ with graphene can yield integrated behavior with novel structural characteristics and dissimilar properties not possessed by the individual components, with improved functional performance with respect to graphene or PtSe_2_ alone (Fig. 4a). By combining their advantages of ultra-high stability and low-energy photon absorption efficiency, PtSe_2_/graphene heterostructures, containing atomically sharp interfaces like traditional metal-semiconductor (M-S) photodiode, exhibit pronounced photovoltaic characteristics (Fig. 4b). Figure 4c shows the linear dynamic range of the device under different bias voltages (linear dynamic range is more than three orders of magnitude). The sensitivity under 0.3 THz laser illuminations (0.08 A/W at unbiased voltage) is competitive with state-of-the-art two-dimensional materials detectors already in the first implementation (Fig. 4c, d). Thus, the superior performance of the PtSe_2_/graphene heterojunction proved in this work reveals its huge potential for broadband low-energy photon detection.

From the perspective of practical applications, noise-equivalent power (NEP) is another key figure of merit to evaluate the performance of a photodetector. Explicitly, NEP is defined as the lowest detectable power in 1 Hz bandwidth. On the low-frequency side, 1/*f* noise (flicker noise) dominates the noise current contribution. The low-frequency flicker noise current, written as *S*_R_/*R*^*2*^ = *α*_H_/*Nf* (where *α*_H_ is Hooge parameter, *S*_R_ is the power spectral density of the resistance fluctuations, *N* is the carrier concentration), originates from the *N* and *µ* fluctuations, reported in two-dimensional materials^44^. Notably, for frequencies beyond 1 kHz, the noise current of the device quickly decreases to the Johnson noise level. To facilitate the applications, we also measure and analyzed the noise spectra of the device as a function of frequency in Figure S8 (Supporting Information). It manifests a structureless broadband noise in 1/*f* at low frequencies, approaching the Johnson-Nyquist noise (*N*_*j*_) limit at higher frequencies. Comparing the measured noise spectral density (NSD) of the three devices, the values of NSD without bias are 7.1 pA/Hz^0.5^ for microgap slit device, 8.7 pA/Hz^0.5^ for nanoslit device, and 3.2 pA/Hz^0.5^ for the PtSe_2_-graphene device, respectively. The NSD of the PtSe_2_-graphene device is the lowest due to the reduction of thermal agitation at the van der Waals heterogeneous interface under dark conditions. We find that the theoretical values via *N*_*j*_^*2*^ = 4*k*_B_*T*/*r* + 2*qI*_d_*r*^2^ for the three devices match with the experimental results, indicating that the main sources of noise are thermal and dark-current noise (for further details see “Materials and methods” and Supporting Information, Figure S8). Thus, the NEP of the heterojunction integration, calculated as NSD/*R*_v_, is then evaluated to be ~38.1 pW/Hz^0.5^, being superior to that of the individual microgap slit device and the nanoslit device (89 pW/Hz^0.5^), and the resulting NEP plots are in Fig. 4e for *V*_bias_ = 0 and 0.1 V, respectively. In terms of (i) speed and (ii) sensitivity, the reporting device demonstrates relatively superior performance as shown in Fig. 4f. Moreover, an order-of-magnitude improvement in sensitivity could be feasible by increasing the absorption strength through, for example, external antenna coupling or nanogap asymmetric channel with more matched heterointerface. For broadband frequencies response in the low-energy photon region, the responsivity is superior to earlier graphene- and black phosphorus-based detectors^32,45^. We, therefore, can affirm that our detector uniquely offers improved performances in terms of (i) speed and (ii) sensitivity in the low-energy photon regime, circumventing drawbacks of current technology regarding expensive costs, cryogenic operation, and low speed of operation.

To exploit the huge potential of low-energy photon processing, imaging technologies in non-invasive inspection methods are crucial. Consequently, to assess the technological potential of the PtSe_2_-based detector, we perform low-energy photon imaging of samples concealed behind opaque objects, i.e., single-view scans of a fresh leaf (Fig. 4g). Compared with the other thermal detector, the PtSe_2_-based detector has the potential for real-time THz imaging. The possibility for the microgap detectors to be configured in arrays is another advantage of PtSe_2_-based detectors for THz imaging applications.

## Discussion

We have demonstrated a novel multilayer PtSe_2_-based low-energy photon detector associated with a bow-tie antenna, which funnels the incident THz electromagnetic wave exactly at the small photoactive area of the detector. The low-energy photon detectors enable room-temperature rectification of a low-energy photon signal, with an ultimate optical responsivity as high as 0.2 A/W (0 V, 0.3 THz) and with a NEP as low as 38 pW/Hz^0.5^. Remarkably, the achieved sensitivity performances are here exploited for nondestructive imaging of macroscopic samples, in a realistic setting. More importantly, we have generated strategies to improve device performance, by implementing optimizations of (i) the antenna, (ii) Ohmic contacts, (iii) van der Waals heterojunctions, and (iv) absorption employing accurately designed subwavelength structures. Nevertheless, further improvement in PtSe_2_-based low-energy photon detector sensitivity can potentially be achieved in the future. Moreover, by using synchrotron-based surface-science techniques, we have validated chemical inertness towards ambient gases (oxygen and water) of PtSe_2_ surfaces and, correspondingly, the long-term stability of PtSe_2_-based devices. Considering the superior ambient stability and the excellent potential for scalable synthesis of PtSe_2_, our work opens new possibilities for the facile realization of portable room-temperature, low-photon detectors, with high sensitivity, fast operation, and low NEP, with great advantages compared to current technologies. Our results represent an important milestone in the roadmap towards the development of high-performance photonic devices, such as low-energy photon cameras, photodetectors, modulators, etc.

## Materials and methods

### Sample growth

Single crystals of PtSe_2_ used in this work were grown by the CuSe flux method^46^. Mixtures of Pt (99.99%) foil, Cu foil (99.99%), and Se ingot (99.999%) in the ratio of 1:6:8 were placed in an alumina crucible with an alumina frit and sealed in an evacuated quartz tube. The quartz tube was heated to 990 °C for 10 h, homogenized at this temperature for 10 h, and then slowly cooled with a rate of 2 °C/h to 660 °C. Subsequently, the quartz tube and its contents were then centrifuged to filter the excess CuSe flux. The plate-like crystals with typical dimensions of about 4 × 4 × 2 mm^3^ were obtained. Figure X1 shows the single crystal XRD pattern at room temperature. One can see that only the (0 0 l) diffraction peaks are present, suggesting that the crystallographic c-axis is perpendicular to the shining surfaces of the samples. Also, the crystallization directions were identified by the Laue diffraction (Photonic Science). In the inset of Fig. S1, we present the Laue diffraction pattern of PtSe_2_ along the [0 0 1] direction, confirming good crystallization of our crystal as judged by the sharp spots in the Laue pattern.

### Synchrotron-based XPS

XPS measurements were carried out on the APE-HE beamline at Elettra Synchrotron in Trieste (Italy). XPS spectra were acquired with an Omicron EA125 hemispherical electron energy analyzer in normal emission configuration, using photon energies ranging between 400 and 900 eV, with an overall energy resolution of about 0.1 eV. Pt-4f core levels were analyzed using Doniach-Šunjić doublet line-shapes, while Se-3d core level was decomposed using Voigt line-shape functions after the subtraction of a Shirley-type background.

### Device manufacturer

PtSe_2_ flakes were exfoliated onto an intrinsic silicon substrate capped with thermally grown SiO_2_ (300 nm). The scotch tape exfoliation was performed in ambient conditions, starting from high-quality crystals of lateral size 1 mm. The drain-source electrodes of PtSe_2_ devices were fabricated by EBL using the PMMA. Angular-dependent Raman spectroscopy was used to identify the direction of the antenna arm. E-beam evaporation was used to deposit Cr/Au (10 nm/60 nm) electrodes.

### Measurements

The near-field was performed with a near SNOM system built based on reflective optics, making the system usable in a broad wavelength range, from visible out into the THz. The neaSNOM integrated THz-TDS based on fs laser-driven semiconductor antenna THz emitter/receiver combination. Generated THz radiation is focused onto a commercial AFM tip (platinum-iridium) using the parabolic mirror, and highly concentrated near-fields are formed at the apex of the tip interacting with the sample and scattering back into the far-field.

The electrical properties of the devices were measured by Semiconductor Parameter Analyzer (B2912A) using variable voltage mode. For photoresponse measurements, data were acquired using a custom-built optical setup. Electrically chopped continuous low-energy photons of a stabilized microwave source (Agilent E8257D) at 0.04 THz connected to a frequency multiplier (VDI WR 9.0) and lock-in (SR830) amplification technique was used to suppress noise and to enhance the detection of ultralow current with high accuracy at 0.12 THz. A multiplier chain (VDI WR 2.8) driven by an IMPATT diode was used to generate a 10 μW/mm^2^ electromagnetic wave at 0.3 THz. The THz wave was TTL modulated in its amplitude (electronically chopped) with a 1 kHz square-wave signal to facilitate the use of lock-in techniques in the presence of dc offset and 1/*f* noise. A commercially calibrated photoconductive THz receiver (TK100) was used. The responsivity, *R*_v_ was extracted from the measured *I*_ph_ as *R*_v_ = *I*_ph_*R*/(*PS*), where *P* is the power density (0.12 THz: *P* = 10 μW/mm^2^) and the *S* is the diffraction-limited area *S* = *S*_λ_ = λ^2^/4. The noise-equivalent power, NEP, was extracted from the formula NEP = *v*_n_/*R*_v_, where *v*_n_ is the root mean square of the noise voltage and *R*_v_ is the voltage responsivity. The theoretical NEP was extracted from the formula NEP = (4*k*_B_*Tr* + 2*qI*_d_*r*^2^)^0.5^/*R*_v_ where *k*_B_ is Boltzmann’s constant, *T* is the temperature of the detectors, *r* is the resistance, *q* is the elementary charge, *I*_d_ is the dark current of the device. All measurements were performed under ambient conditions. Noise measurements were performed at room temperature in a Lakeshore cryogenic probe station with micromanipulation probes.

## Supplementary information

Supporting Information
