# Supplementary material for: Hybrid Dirac semimetal-based photodetector with efficient low-energy photon harvesting
Source: Light Sci Appl. 2022 Mar 10;11:53. doi: 10.1038/s41377-022-00741-8 (PMC8913679; doi:10.1038/s41377-022-00741-8)
Supplement: Supplementary file 1 — Supporting Information [file 41377_2022_741_MOESM1_ESM.docx]

**Supplementary Information for**

**Hybrid Dirac Semimetal-based Photodetector with Efficient Low-Energy Photon Harvesting**

Lin Wang^1, 2, 9^*, Li Han^1,2^, Wanlong Guo^1,3^, Libo Zhang^1,2^, Chenyu Yao^1^, Zhiqingzi Chen^1^, Yulu Chen^4^, Cheng Guo^5^, Kaixuan Zhang^2^, Chia-Nung Kuo^6^, Chin Shan Lue^6^, Antonio Politano^7,8^*, Huaizhong Xing^2^, Mengjie Jiang^2^, Xianbin Yu^5^, Xiaoshuang Chen^1,3,9^, Wei Lu^1,3^

^1^State Key Laboratory for Infrared Physics, Shanghai Institute of Technical Physics, Chinese Academy of Sciences, 500 Yu-tian Road, Shanghai 200083, China.

^2^Department of Optoelectronic Science and Engineering, Donghua University, Shanghai 201620, China.

^3^School of Physical Science and Technology, ShanghaiTech University, Shanghai 201210, China.

^4^The 50th Research Institute of China Electronics Technology Group, Shanghai, 200331 China

^5^ Research Center for Intelligent Network, Zhejiang Lab, Hangzhou, 311121, China.

^6^Department of Physics, Cheng Kung University, 1 Ta-Hsueh Road, 70101 Tainan, Taiwan, China.

^7^ INSTM and Department of Physical and Chemical Sciences, University of L’Aquila, via Vetoio, 67100 L’Aquila (AQ), Italy.

^8^CNR-IMM Istituto per la Microelettronica e Microsistemi, VIII strada 5, I-95121 Catania, Italy.

^9^College of Physics and Optoelectronic Engineering, Hangzhou Institute for Advanced Study, University of Chinese Academy of Sciences, No. 1, Sub-Lane Xiangshan, Xihu District, Hangzhou 310024, China.

*E-mail: *wanglin@mail.sitp.ac.cn; antonio.politano@univaq.it*

**Section 1 The grown and characterization of PtSe_2_**

**Material grown**

The chemical vapor transport method (CVT) is generally used for the preparation of TMDs materials because of their high melting points and the high vapor pressure from chalcogen components. However, the Iodide element which is commonly used as the transport agent is ineffective for the CVT growth of PtSe_2_ crystals ^[1]^. On the other hand, the CVT growth of PtSe_2_ can be achieved using SeBr4 as the transport agent^[2]^, but the entire process is time-consuming and the hydrolysis of SeBr_4_ decreases the reproducibility.

**Material characterization**

**AFM:** Atomic force microscopy (AFM) was used to probe the detailed surface morphology and the thicknesses of PtSe_2_ sheets. From the high-resolution optical microscopy images, we can observe that the PtSe_2_ sheets are well-defined which is attributed to the homogeneous thickness of PtSe_2_ sheets. The atomic force microscopy (AFM) measurements were performed by Bruker Dimension Edge using tapping mode.

**Raman:** Raman spectroscopy was utilized to identify and characterize the obtained PtSe_2_ films using a 633 nm laser at a power of <300 μW. The spectra shown for each sample were obtained by averaging 10 discrete point spectra. The peak centered at 177 cm^−1^ can be assigned to the E_g_ in-plane vibrational mode of Se atoms and the peak centered at 206 cm^−1^ to the A*_1g_* out-of-plane vibration mode. Raman spectroscopy was carried out using HORIBA Lab Ram HR800 Raman system. The spectra were calibrated by 520.7 cm^-1^ phonon mode from the silicon substrate.

**TEM:** To further assess the microstructure, crystallinity, and elemental composition of the PtSe_2_ materials, the samples were transferred onto a copper grid using the polymethyl methacrylate) (PMMA) assisted transfer method and investigated by TEM. The low-magnification TEM image (Fig.S1e) indicates that the PtSe_2_ nanosheets have good uniformity and continuity across the whole platelet. Fig. S1f shows the selected area electron diffraction pattern of PtSe_2_ nanosheet, which confirms the obtained sample is polycrystalline. The polycrystal PtSe_2_ layered film further confirms a few nanometer-scale domain sizes in diameter, which is also similar to what has been observed for the selenization of WSe_2_. TEM characterization was performed using a JEM-200CX, operating at 200 kV, and equipped with an EDS system.

**XPS:** Figure S1g shows the single crystal XRD pattern at room temperature. Only the (0 0 l) diffraction peaks are present, suggesting that the crystallographic c-axis is perpendicular to the surfaces of the samples. The crystallization directions were identified by the Laue diffraction (Photonic Science). In the inset of Fig.S1e, we present the Laue diffraction pattern of PtSe_2_ along the [0 0 1] direction, confirming good crystallization of our crystal as judged by the sharp spots in the Laue pattern. Pt-4f and Se-3d core levels are split into two components with Pt-4f_7/2_ and Se-3d_5/2_ components at 73.4 and 54.7 eV, congruently with previous reports for the same system ^[3]^. Figure S1h shows that the material has good stability.

**
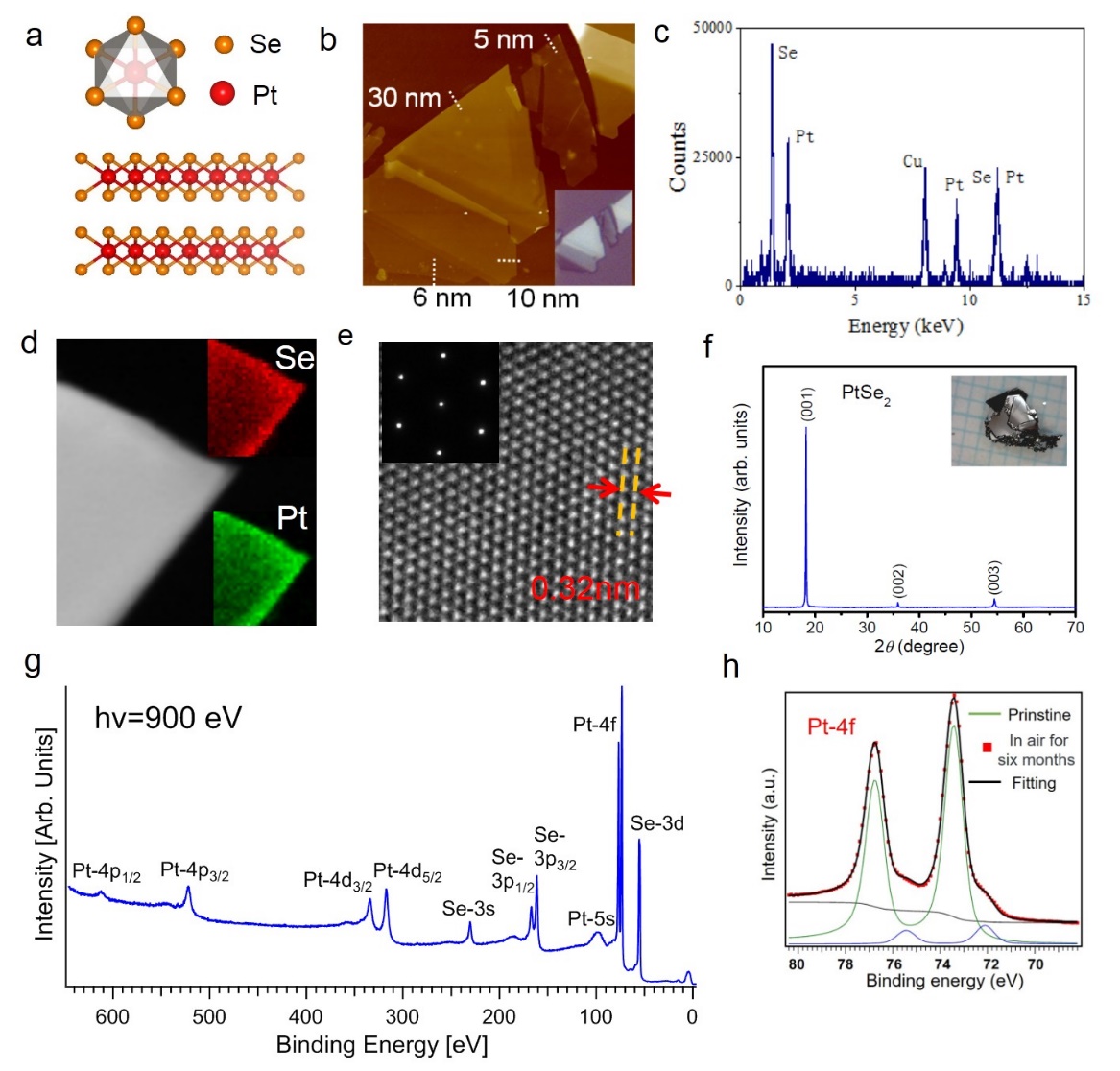
**

Figure S1. Characterization of PtSe_2_ crystals. The crystal structures of PtSe_2_, PtSe_2_ is a layered transition-metal dichalcogenide crystallized in 1T structure, each layer has a Se-Pt-Se sandwich structure with the center Pt octahedrally surrounded by Se atoms. Gray balls are Pt atoms, and green balls are Se atoms.b Optical microscope image (the inset) and atomic force microscope (AFM) image of the PtSe_2_ flakes. The thickness of the exfoliated PtSe_2_ flakes in this work is confirmed to be around 6-30 nm. c EDS of the PtSe_2_ flakes, with the photo of grown single crystals in the inset. The signal of the Cu element comes from the supporting TEM grid. d the right pictures are the corresponding Pt and Se elemental maps from the triangular region in the left picture. The uniformity and crystallinity of the PtSe_2_ flake are validated by energy-dispersive X-ray spectroscopy (EDS) mapping images along with the high-resolution transmission electron microscopy (HRTEM) image. e Transmission electron microscopy characterization of the exfoliated PtSe_2_ few layers. f The X-ray diffraction of pristine PtSe_2_. g Survey X-ray spectrum of as-cleaved PtSe_2_ with X-ray photons of 900 eV. Only Pt- and Se-derived core levels were recorded. No traces of contaminants were recorded. h Pt-4f core levels measured at a photon energy of 400 eV.

**Section2 Electric characterization of PtSe_2_ Photodetector**

We prepared a PtSe_2_ back-gate photodetector. The device is shown in Figure S2 a. The resistance is 3.6×10^5^ Ω. Figure S2 b is the transfer characteristic of the PtSe_2_ device. Based on this, we calculated the carrier mobility of PtSe_2_ at 965 cm^2^ (V·s)^-1^ (µ = $g_{m}\cdot\frac{L}{W}\cdot\frac{1}{\text{C}\text{OX}\text{V}\text{SD}\text{ }}$ where *L* and *W* are the channel length (6μm) and width (4μm), respectively, *C*_ox_ is the oxide capacitance per unit area (11.5 nF cm^-2^), and g_m_=$\frac{\text{d}\text{I}\text{SD}}{\text{d}\text{V}\text{G}}$=740 nA V^-1^.) We also measured the photoresponse of the device under back-gate voltage. As shown in Figure S2 c, the device with a thickness of about 5.6 nm has almost no response in the terahertz band.


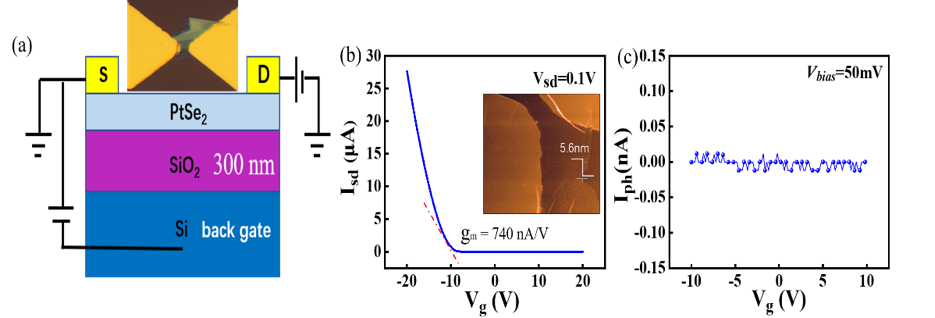


Figure S2 a. Structure diagram of PtSe_2_ device. b. Transfer characteristic of the PtSe_2_ device. c. The photoresponse of the device under the gate voltage.

**Section3 Nonequilibrium states driven photoresponse under the applied static electric field**

For a typical device with symmetrical contacting or material geometry, the photocurrent is absent without bias voltage traversing across the channel. By applying voltage, the asymmetry should arise due to the potential gradient or change of quasi-Fermi level across the channel. The nonequilibrium carriers are strongly excited at material-contact edges, while the flow of nonequilibrium hot carriers are ideally compensated each other when the voltage is zero as shown in Fig. S3(1) a. The Seeback coefficient is following the formula[4]:

$$\text{S≈}\frac{\text{2}\text{π}^{\text{2}}}{\text{3}}\frac{\text{k}_{\text{B}}^{\text{2}}\text{T}}{\left| \text{e} \right|\text{μ}}$$

Instead of that, the unilateral flow of nonequilibrium carriers can be given rise due to the bias-induced asymmetry of quasi-chemical potential as shown in Fig. S3(1) b. A similar process is demonstrated in Ref. [5]


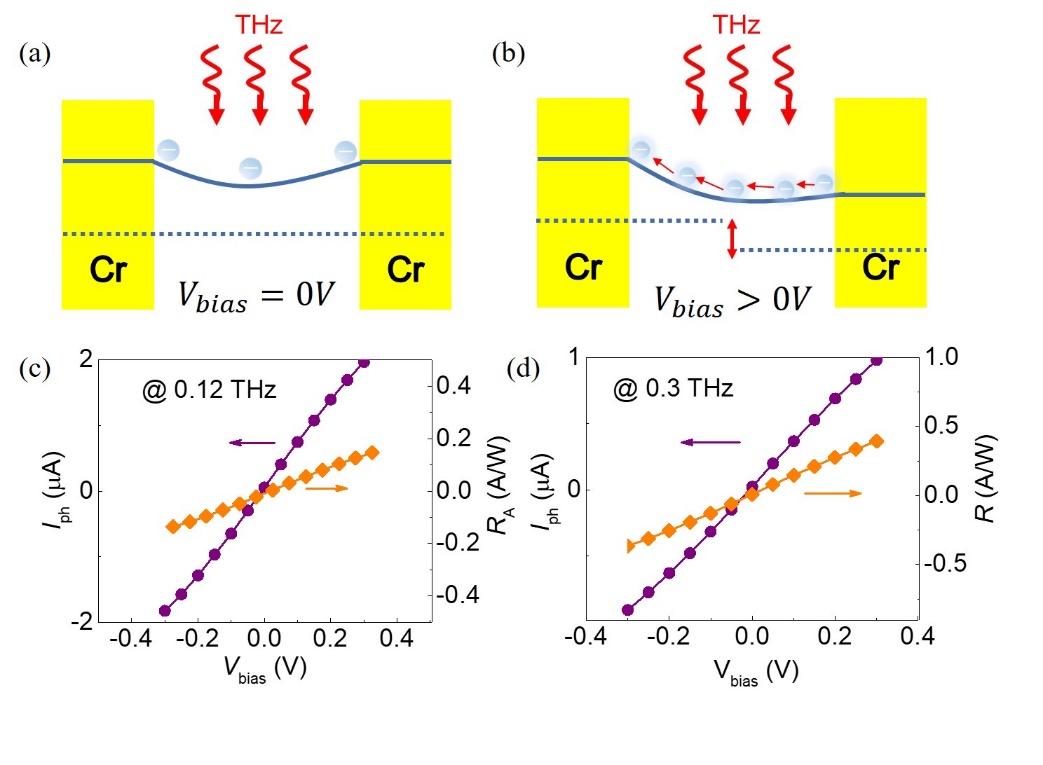


Figure S3 (1) a-b. Schematic diagram of photoresponse under the applied static electric field.

c-d. Bias dependence of responsivity at 0.12 THz and 0.3 THz

Figure S3 (2) shows the I-V characteristics of the device at different temperatures. As the temperature increases, the resistance becomes larger. According to Figure S3 (2) b-c, the output current increases significantly under THz radiation when the device is biased at different positive or negative voltages.


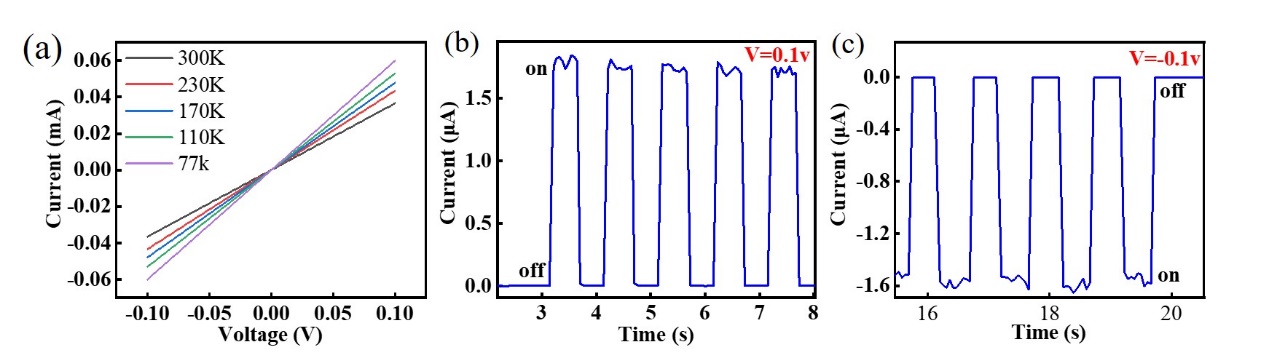


Figure S3 (2) a. The I-V characteristics of the device at different temperatures. b-c. The photoresponse of the PtSe_2_ device under positive and negative 0.1V.

**Section 4 The microgap slit device in bias mode**

To gain further insight into the above bias-induced nonequilibrium mechanism of PtSe_2_-based device, we fabricated four-terminal detectors by using PtSe_2_ nanosheets. In addition to the bow-tie shape antenna-electrodes, there are additional two contact fingers with orientation perpendicular to the direction of the antenna. In this regard, the photo signal can be readout from two fingers. As expected, the test results also supported the conclusions of the main text. Bias induces the directional movement of non-equilibrium carriers by breaking further the symmetry across the channel and giving rise to the photo signal. From the data, it could be inferred that the signal from two fingers is maximized when the light polarization is along the direction of the bow-tie antenna (Figure S4 a). This is because the localized field near the contact-material interface is largest when the light-polarization is along with the bow-tie antenna. By applying bias-voltage, the photo signals from two fingers changes with the change of voltage. The saturation behavior at larger voltage shown in Fig. S4c and Fig. S4d may be caused by the electron flow interfered by adjacent electrodes, the change of potential barrier in the middle of the channel at larger voltage.


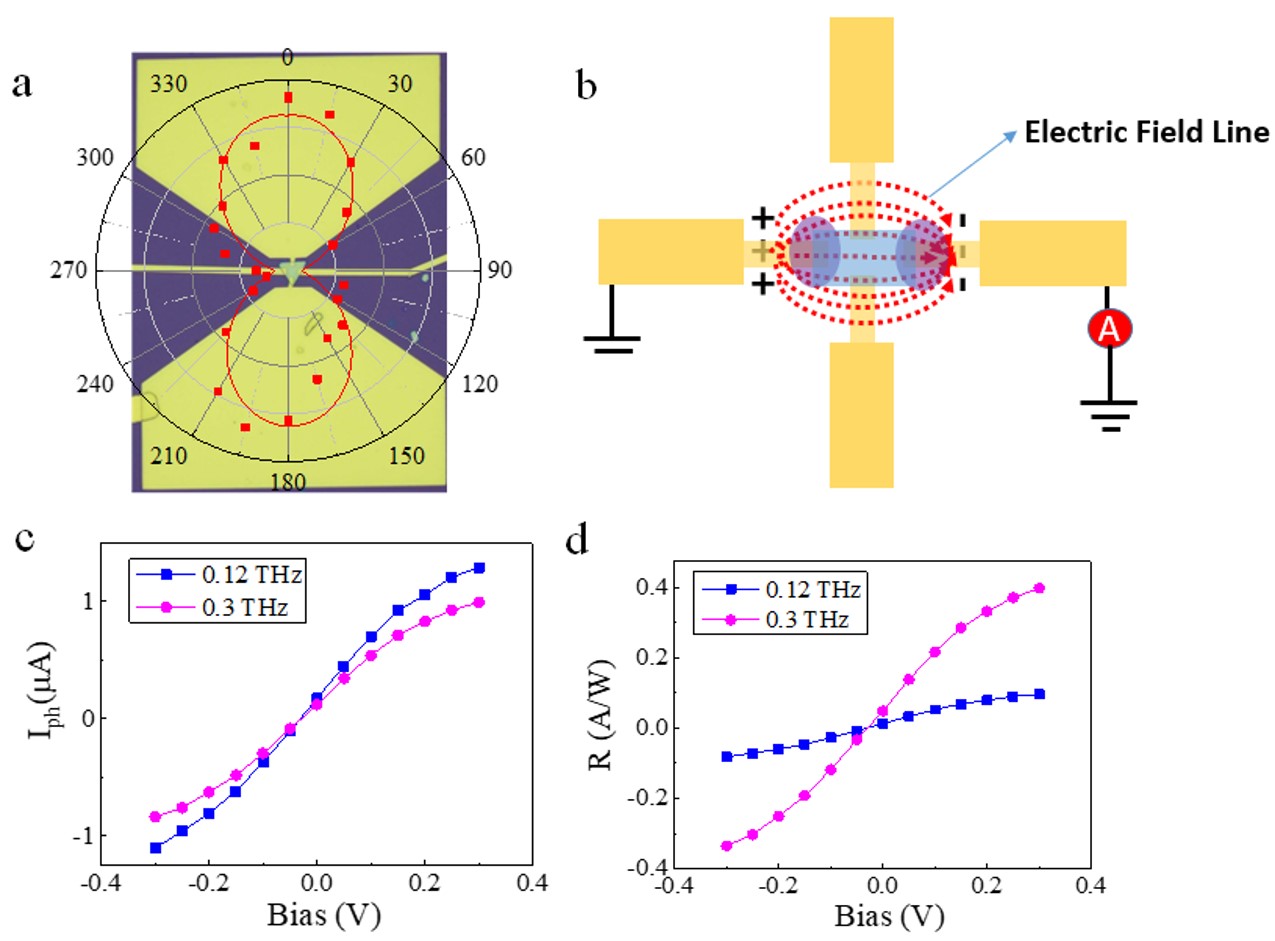


Figure S4 The performance of the four-terminal device. a. The polarization-resolved detection measurements are based on the four-terminal device. b. Nonlinear schematic diagram based on four-terminal structure. c-d. The photoresponse and responsivity of the device under different biases at 0.12 and 0.3 THz.

**Section 5 The** **performance of microgap slit PtSe_2_ device**

The polarization-resolved detection measurements with different illumination geometries allow detailed and quantitative analysis of various factors contributing to the overall detector performance. Concerned about this, we measured the devices under normal illumination at 0.3 THz with its electric vector along the variable angle concerning the antenna axis showing how the antenna improves the detector's sensitivity. The responsivity is smallest when the low-energy photon electric field is parallel to the slit (Fig. S5e). Due to the strong focusing of the localized electric field, the signal is enhanced when the low energy photon electric field is perpendicular to the slit by exciting the ac charge oscillation, ultimately resulting in the lobe-like polarization diagram.


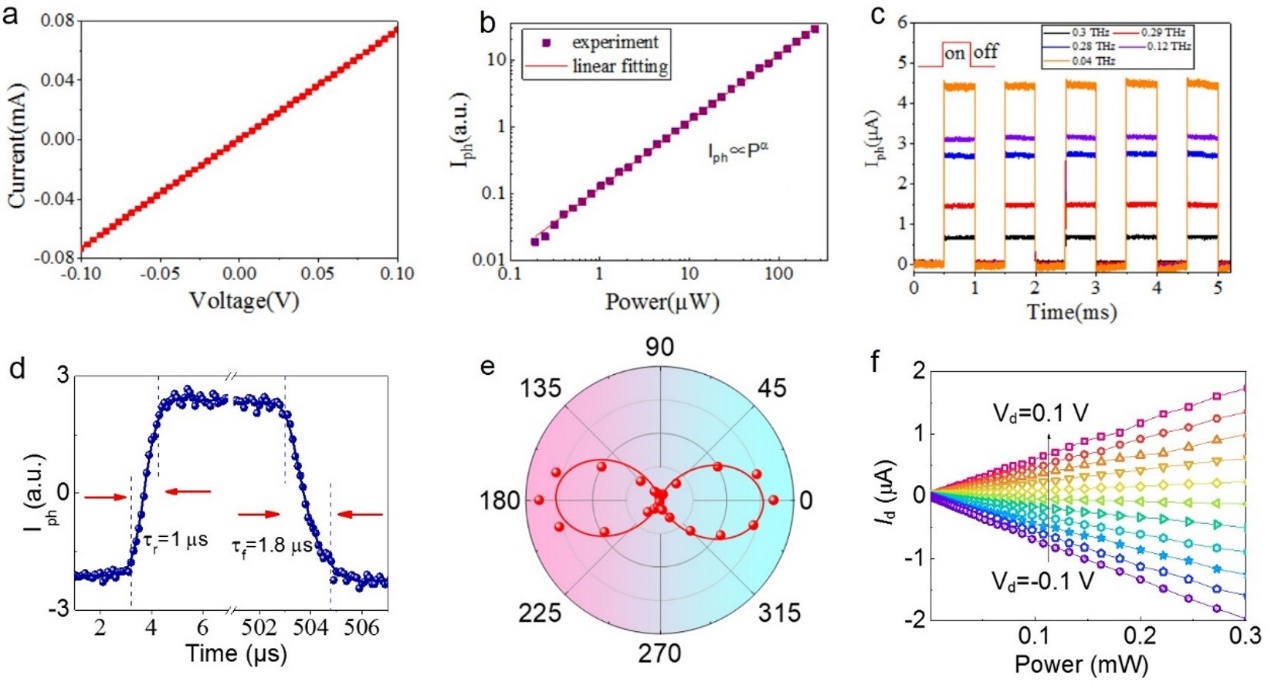
 Figure S5 a. The current-voltage characteristic of the device. b The measured photocurrent vs. output power P for 0.04 THz, with the power varied from 0.3 μW to 300 μW. All the curves are fitted well by a simple power-law *I*_ph_ ∝ *P*^α^ and α > 0.96 can be retrieved. This phenomenon indicates that the photocurrent depends linearly on the incident power over a large range of more than three orders of magnitude. c. The pulsed current response at various excitation frequencies, showing the broadband nature of the PC effect-dominated mechanism. d. The response time measurement. e. The polarization-resolved detection measurements. f. The output power-dependence photocurrent measurements are based on different biases.

**Section 6 The performance of and principal analysis of nanoslit device**

For PtSe_2_, only the top layer can be hybridized by metal top contacts, and thus only the vdW gap between the metal and the top layer of the PtSe_2_ was eliminated. The vdW gaps between the bottom layers still exist. Hence, to improve the carrier injection into the bottom layers, it is preferable to use a few layers of PtSe_2_. Although several selected metals form strong hybridization at interfaces, therefore suppressing the tunnel barrier, Fermi level pinning occurs, due to the work-function change at the metal-PtSe_2_ interface, as well as the creation of gap states from the weakened intralayer Pt-Se bonding. Such effects can significantly impact the work function of metal-doped PtSe_2_. Thus, one can improve the responsivity by applying a bias within the photocurrent generation path. Fig. S6d, e presents the drain-source bias (*V*_Bias_) dependence of the detector current. *I*_ph_ increases for positive *V*_Bias_ (applied to the titanium electrode; chromium electrode grounded), but it decreases for V= 0 V and changes sign at *V*_Bias_=-0.12 V. All curves cross at *V*_Bias_= -0.12 V, that is, for this bias the photocurrent turns to zero regardless of the power of optical illumination. This behavior indicates that the work-function within the PtSe_2_ channel can be influenced by applying a source-drain bias. Fig. S6c schematically shows the band profiles for three different biases. In this case, as a result of the different doping introduced by titanium and chromium, a potential drop occurs within the PtSe_2_ channel. The right panel shows the case for *V*_Bias_>0. Here, the external bias enhances the potential profile within the channel, and the photocurrent increases. In contrast, at V*_Bias_*= -0.12 V (left panel), the external bias just offsets the built-in potential profile, making the band profile flat and the photocurrent zero. Therefore, we estimate, for the PtSe_2_-based device, that the height of the potential within the channel induced by the doping of titanium and chromium is 0.12 eV.

**
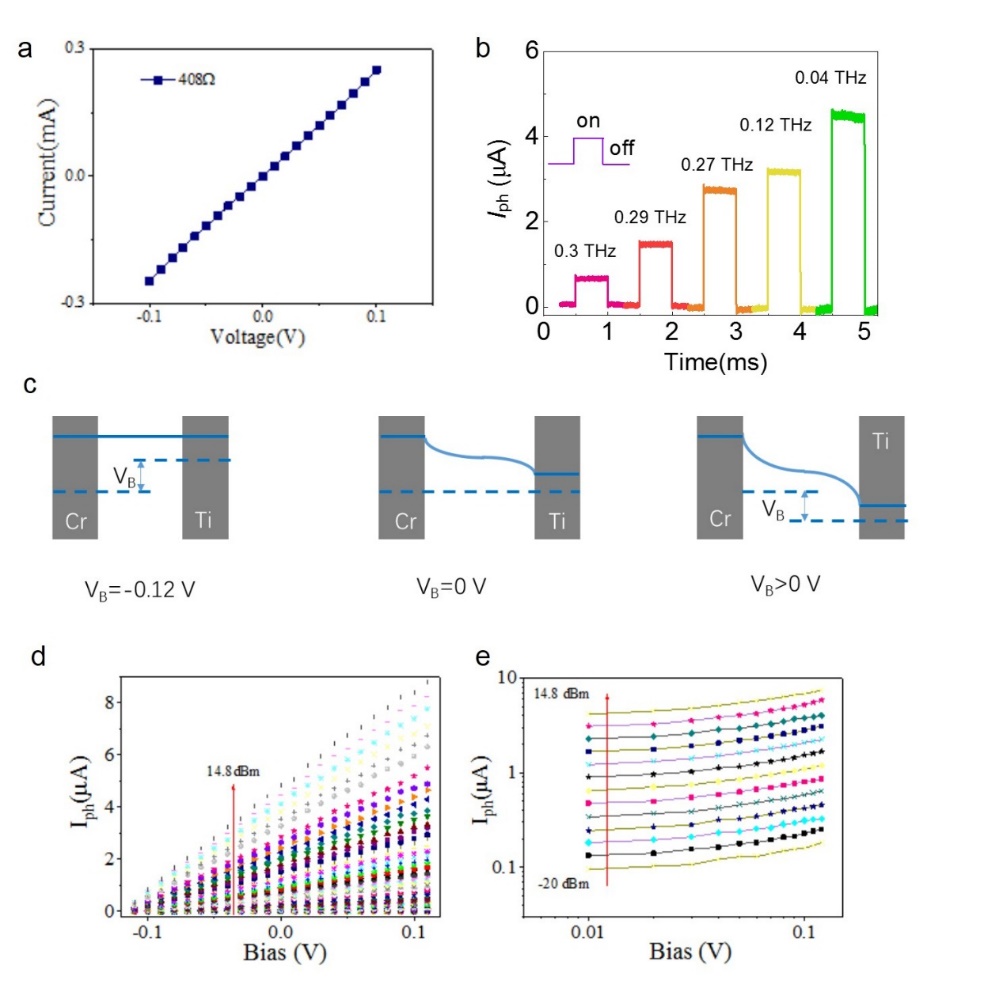
**

Figure S6 The sub-30 nm channel asymmetric device. a. The current-voltage characteristic of the device. b. The pulsed current response at various excitation frequencies. c. The PtSe_2_ band profiles (blue lines) at source-drain biases (V*_B_*) of -120 mV, 0, and positive bias, from left to right, respectively. Source-drain bias (V*_B_*) equals the difference in Fermi level between the chromium- and titanium-doped PtSe_2_. The dotted line represents the Fermi level. d-e. Source-drain bias (V*_B_*) dependence of the M_Cr_-PtSe_2_-M_Ti_ photoresponse. Current versus source-drain bias (V*_B_*) with and without light illumination, an excitation wavelength of 1 mm. right: measured external photocurrent of the M_Cr_-PtSe_2_-M_Ti_ photodetector as a function of the source-drain bias V*_B_*>0. The curve denotes the magnitude of the photocurrent at an incident power from -20 dBm to 14.8 dBm.

**Section 7 Analyze and characterize the stability of materials through XPS measurement**

Both Pt-4f and Se-3d core levels did not exhibit changes upon gas dosage. Especially, the SeO_2_ component at ~59 eV^[6]^ is absent, thus proving surface stability even in an oxidative environment. Such a value of the binding energy was previously reported for Pt-based organometallic compounds ^[7]^ and chemisorbed CO ^[8]^, as also supported by the appearance of C-1s signal in XPS. To unveil the origin of the additional doublet, we probed the vibrational spectrum by high-resolution electron energy loss spectroscopy (HREEELS). The observation of intense C-H bending and stretching vibrations at 180 and 360 meV in air-exposed samples (Fig. S7) unambiguously indicate adsorption of airborne CHx contamination ^[9]^, whose surface coverage is just 0.1 ML, with ML being monolayer. The adsorption of this insignificant coverage of CHx only occurs at Se vacancies of the outermost surface layer, forming a chemical bond with Pt atoms, as evidenced by the analysis of the Pt-4f core level. Notably, even after air exposure, we did not observe in XPS spectral contributions from PtOx species, characterized by Pt-4f_7/2_ at a binding energy of ~74 eV, in contrast with other reports in the literature ^[10]^. The reduced amount of Se vacancies in our single crystals drastically increases the oxidation resistance of the samples. The valence band is substantially unchanged upon gas dosage. On the other hand, air exposure progressively introduces a broadening of valence-band features, although overall changes remain negligible, especially near the Fermi level. A careful inspection reveals that the density of states around Fermi level only slightly increases with aging in air, due to contributions from CHx-derived electronic states.


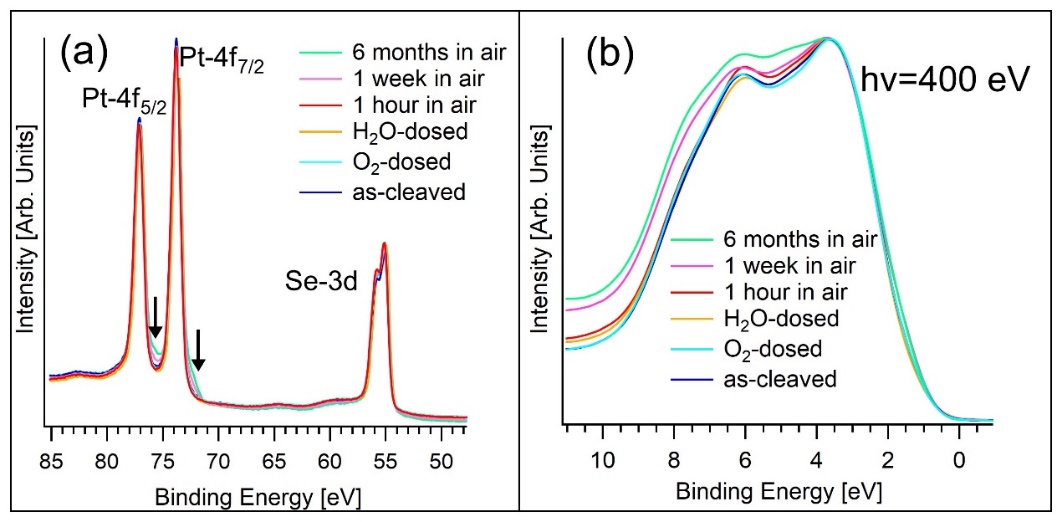


Figure S7: a. Pt-4f and Se-3d core levels and b the corresponding valence band of the as-cleaved PtSe_2_ surface and its modification upon the dosage of 10^6^ L of O_2_ and H_2_O. The same surface was kept in the air for different periods, up to six months. The photon energy in all spectra is 400 eV.

**Section 8 Corresponding noise current density for three devices**


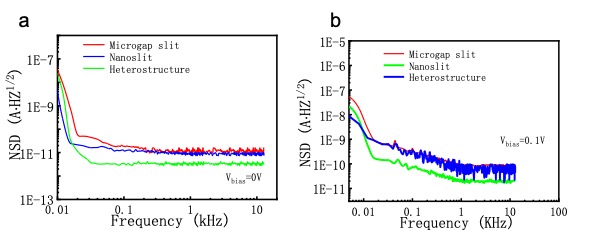


Figure S8 a-b Noise current density as a function of modulation frequency for the PtSe_2_ device at zero bias and 0.1 V using amplifiers and a dynamic signal analyzer SR785.

**Section 9 Imaging**

We verified the usefulness of the PtSe2-based detector by demonstrating its applicability to imaging. The sample to be imaged is a fresh leaf, as shown in Fig. 3g, is attached on optically transparent tape. The sample was mounted on an x-y stage and scanned through the position slightly behind focus while the PtSe_2_-based detector was aligned at the focus. An image of the fresh leaf sample radiated by the THz source, acquired by PtSe_2_-based detection. The THz beam scattered on the boundary results in a lower energy density. This small variation can be detected and distinguished by the PtSe_2_-based detector, demonstrating the sensitivity and unique detection mechanism of the PtSe_2_-based detector platform. The resolution for both images was determined by the scanning step size, which was 1 mm in each direction. Fig. 3g shows the THz image in one dimension acquired by the PtSe_2_-based detector with a scanning step size of 0.4 mm along the y-direction.

The speed of single-pixel-based raster-scanning imaging is restricted to the speed of the stepping motor and we have reduced the lock-in time constant. As shown in the figure, different lock-in time constants have no effect on the imaging results. The lock-in integration time is set to a constant, respectively. (10 ms,100 ms, 300 ms). This shows that the SNR of the imaging system is minimally affected by changing the lock-In time constant from hundreds ms down to 10 ms.


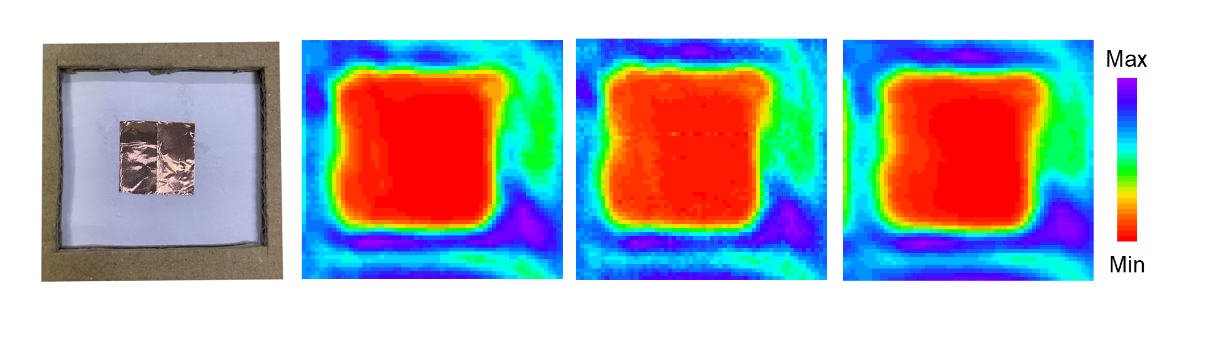


Figure S9 The single-pixel-based raster-scanning imaging

**Section 10 Simulations**

To clarify the influence of nanoslit on the field enhancement effect, we used FDTD to simulate the field distribution in the structure. In the simulations, all types of incident sources are selected as a plane wave, which illuminates the structure vertically. The boundary condition is selected as a perfect matching layer (PML), and the distance between it and the structure should not be too close to prevent the echo from affecting the simulation results. Since the plasma frequencies of metals are in the visible and near-infrared, for terahertz and microwave frequency, metals are generally modeled as perfect electrical conductors (PEC) ^[11]^. The antenna thickness is set to 70nm. In addition, as shown in Figure S10, by simulating the field distribution under different thicknesses, it is obtained that the electric field intensity is not affected by it. In this simulation, we recorded the electric field value of antennas with different metal thicknesses through the monitor (100 nm channel, 0.3 THz). THz spoof-plasmon polaritons can be efficiently launched to convert an incident electromagnetic field into the localized oscillating electric field in the bow-tie antenna with a subwavelength gap (see figure S10 a). as shown in figure S10 b below, It can be seen that the change in thickness does not affect the electric field.


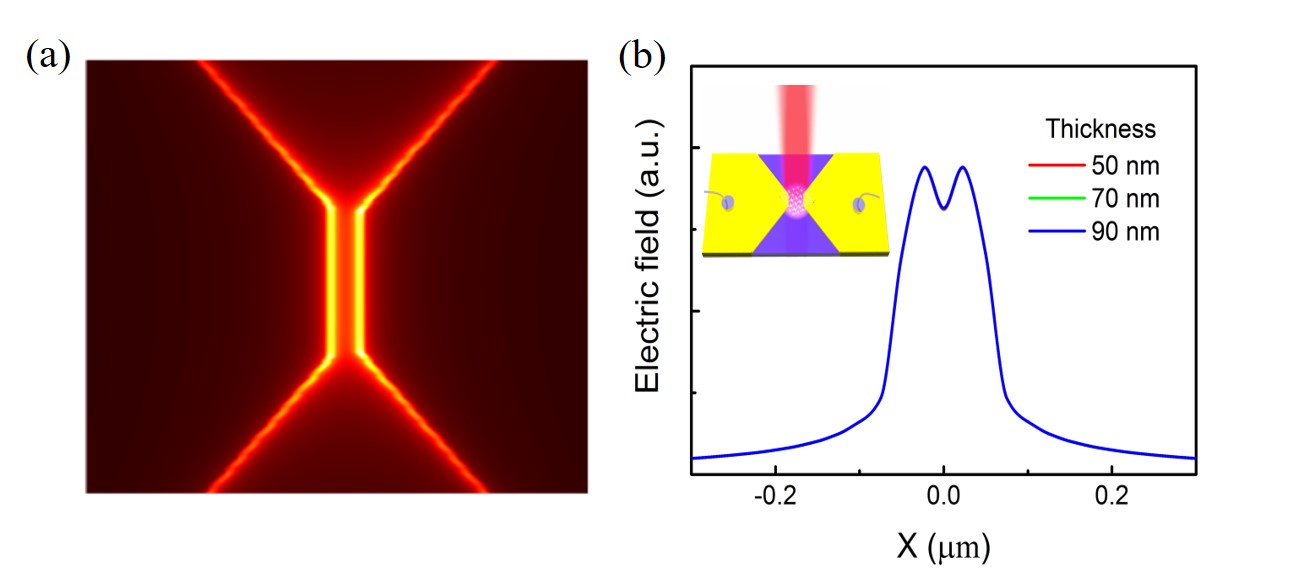


Figure S10 The effect of antenna thickness on the electric field. (a) Localized oscillating electric field enhancement in the channel. (b) The thickness of the antenna has such a small effect on the electric field that the curves coincide completely.

**Reference**

[1]Hu, D., Zhao, T., Ping, X., Zheng, H., Xing, L., Liu, X., Zheng, J., Sun, L., Gu, L., Tao, C., Wang, D. and Jiao, L. Unveiling the Layer-Dependent Catalytic Activity of PtSe2 Atomic Crystals for the Hydrogen Evolution Reaction. Angewandte Chemie 58, 6977-6981 (2019).

[2]Zhang, K., Yan, M., Zhang, H., Huang, H., Arita, M., Sun, Z., Duan, W., Wu, Y. and Zhou, S. Experimental evidence for type-II Dirac semimetal in PtSe2. Physical Review B 96, 125102, (2017).

[3]Wang, Z., Li, Q., Besenbacher, F. and Dong, M. Facile Synthesis of Single Crystal PtSe2 Nanosheets for Nanoscale Electronics. Adv. Mater. 28, 10224-10229, (2016).

[4] Fereshte, G., Hong-Yi, X., Takashi, T., Kenji, W., Matthew, S., and Philip, K. Enhanced Thermoelectric Power in Graphene: Violation of the Mott Relation by Inelastic Scattering. Phys. Riew. Lett. 116, 136802 (2016)

[5]Tong, J., Zhou, W., Qu, Y., Xu, Z., Huang, Z., Zhang, D. H. Surface plasmon induced direct detection of long wavelength photons. Nature Communications, 8, 1660 (2017).

[6]Bachvarova-Nedelcheva, A., Iordanova, R., Kostov, K. L., Yordanov, S. and Ganev, V. Structure and properties of a non-traditional glass containing TeO_2_, SeO2 and MoO_3_. Optical Materials 34, 1781-1787, (2012).

[7]Hanks, T., Ekeland, R., Emerson, K., Larsen, R. and Jennings, P. Reactions of diazomethane derivatives with platinum (II): a facile method for platinum ylide preparation. Organometallics 6, 28-32, (1987).

[8]Shimizu, S., Noritake, H., Koitaya, T., Mukai, K., Yoshimoto, S. and Yoshinobu, J. Site-specific chemical states of adsorbed CO on Pt (997): A high resolution XPS study. Surf. Sci. 608, 220-225, (2013).

[9]Li, Z., Wang, Y., Kozbial, A., Shenoy, G., Zhou, F., McGinley, R., Ireland, P., Morganstein, B., Kunkel, A., Surwade, S. P., Li, L. and Liu, H. Effect of airborne contaminants on the wettability of supported graphene and graphite. Nat. Mater. 12, 925-931, (2013).

[10]O’Brien, M., McEvoy, N., Motta, C., Zheng, J.-Y., Berner, N. C., Kotakoski, J., Elibol, K., Pennycook, T. J., Meyer, J. C. and Yim, C. Raman characterization of platinum diselenide thin films. 2D Mater. 3, 021004, (2016).

[11]Maier, S. A. Plasmonics : fundamentals and applications. Springer Berlin (2007).
